# Portable TD-OCT Scanner for Dental Use

**DOI:** 10.3390/bioengineering13070743

**Published:** 2026-06-25

**Authors:** Tatsuo Shiina, Seiroh Okaneya

**Affiliations:** 1 Graduate School of Engineering, Chiba University, 1-33 Yayoi-cho, Inage-ku, Chiba-shi 263-8522, Chiba, Japan; 2 Phoenix-Dent Co., Ltd., 2649-3 Kamei-cho, Sano-shi 327-0024, Tochigi, Japan; seiroh@phoenix-dent.co.jp

**Keywords:** OCT, dental, portable, teeth, gum, caries

## Abstract

A portable OCT scanner usable for dental applications is designed to be compact (B4 notebook size), lightweight, and capable of DC battery operation, enabling chairside use and mobility in home medical care. TD-OCT faces the challenge of a slower measurement speed compared to SD/SS-OCT. In this study, by utilizing multiple rotating reflectors and combining them with a 3 × 3 fiber coupler, a measurement range of over 10 mm and a measurement speed of 1000 scans per second were achieved. Additionally, the dental intraoral probe was designed to ensure high accessibility, allowing free positioning within the oral cavity, with an ingeniously compact optical system. A cylindrical probe with a diameter of 6 mmϕ and a length of 10 mm was developed to enable tooth measurement. This study demonstrated the capability to evaluate attenuation coefficients derived from material properties, starting with measurements of caries and gums.

## 1. Introduction

The current state of dental diagnosis begins with visual inspection, followed by tactile examination using probes, X-ray imaging devices, and, more recently, the introduction of CT scans. On the other hand, gums are diagnosed for periodontal disease and gingival recession also through visual and tactile examinations. The challenge lies in the variability of judgments due to the subjective diagnosis by physicians. Additionally, there is significant variation in the physicians’ diagnoses themselves, resulting in outcomes dependent on their skill and experience [1].

Today, optical devices are being introduced from the perspectives of objectivity in diagnosis and teeth measurement, as well as ease of judgment. Optical caries detection devices support the diagnosis of caries and quantify the extent of decay. They irradiate laser light onto the lesion, receive the fluorescent reflection coaxially, and display the degree of caries numerically. Products such as Diagnodent (Kavo Dental) have been commercialized [2,3]. For application in implant procedures, 3D shape measurement devices have also been introduced [4,5]. They are used for acquiring highly accurate 3D shape data and evaluating surface texture. Traditional materials like silicone used for dental impressions require placement in the mouth and waiting for them to solidify, but optical measurement of the oral cavity, including teeth, supports high-precision and rapid diagnosis [6,7,8].

Furthermore, there are high expectations for Optical Coherence Tomography (OCT), which began with reports of cross-sectional measurements inside teeth, and has since been widely reported for caries, microcracks, and measurements of the enamel–dentin junction (EDJ) [9,10,11,12,13,14,15]. Unlike X-rays, there is no concern about radiation exposure, and sufficient resolution when addressing the aforementioned challenges is being discussed. Dental OCT devices have also been reported, with examples of their use shown. Groups led by Sumi and Shimada have reported on the development and clinical application of dental OCT [9,11,14,16,17,18,19,20,21]. They are examining the realization and clinical application potential of dental OCT. They report plural OCT data with sufficient quality, and thorough discussions are held from various perspectives, while their challenges include the large size of the devices themselves, requiring patients to bring the affected area to the device. Moreover, the probes are large, limiting the freedom of measurement within the oral cavity.

In this study, based on the above background, the development of a portable OCT scanner is underway. It is intended for chairside use. Moreover, it can be battery-operated, making it suitable for use in home healthcare visits. The device is designed to be easy to use by anyone, allowing measurement to start with a single push of a button. Additionally, it is designed with expandability in mind, not limiting the measurement target to caries alone, but also including periodontal disease and other oral diseases [22,23].

Specifically, Time-Domain OCT (TD-OCT) is employed in this study. For speeding up the process, a method using a combination of a rotating reflector and a fixed mirror is used for the optical path scanning mechanism [24]. The system incorporates a 3 × 3 optical fiber coupler to create a proprietary optical system, too. Furthermore, waveform processing is performed directly in hardware using a custom high-frequency circuit. This achieves a measurement speed of up to 1000 scans per second. Since data with depth information is streamed to a PC, image processing on a general PC or a tablet is easy. Additionally, the probe is quite small, and it ensures high accessibility, allowing free positioning within the oral cavity, and features a highly compact optical system. A cylindrical probe with a diameter of 10 mm was developed, and after several designs, a final cylindrical probe with a diameter of 6 mm and a length of under 10 mm was developed. Using these optical probes, various measurement cases of teeth and gums were considered.

This report presents the design and development of the portable dental OCT scanner. In device development, the measurement target is not limited to teeth, and the design anticipates future speed improvements and multifunctionality depending on the usage environment in oral care and disease measurement. Measurement cases are shown, and future developments regarding speed enhancement and expandability are discussed. Ideas related to oral cancer and periodontal pocket measurement are also mentioned.

## 2. Method

### 2.1. Portable TD-OCT Scanner

The development of OCT began with TD-OCT and has evolved and advanced to FD-OCT and SS-OCT [25,26,27,28]. Especially for ophthalmic OCT, development has progressed with the aim of performing measurements faster and with higher resolution to reduce image blurring caused by pulsation of the measurement target due to heartbeat, breathing, and pupil movement. Depth scanning is performed by electrical scanning, enabling high-speed measurement with speeds of 10k–100k scans/s and high resolution of 3–5 μm. On the other hand, due to the high resolution, the device’s casing and stand must have high rigidity, making the device large and immovable, and the measurement style involves bringing the part to be measured in front of the device. The measurement range, speed, and resolution are uniformly determined by the selection of the light source. Since the measurement waveform is processed by FFT calculation to obtain depth information, the computational load is high. Fiber-based small optical probes for OCT are reported, while they have some restrictions on A-scan depth, flexibility, and so on [29,30,31]. Oral soft tissue imaging has been successfully reported, while it is difficult to make the measurement probe small enough for the use of freely positioning inside the oral cavity [32,33]. For dental applications, it is desirable for the device to be chairside and freely movable to observe the oral cavity in any posture. The measurement probe must be able to freely access the oral cavity and allow for flexible positioning. Both the device and the measurement probe need to be compact.

This study adopts TD-OCT. The reason is the high design flexibility. The light source selection, measurement range, speed, and optical probe can be designed independently. In SS-OCT, the imaging depth is determined by the wavelength scanning range of the swept source and generally does not exceed 10 mm. TD-OCT has no such constraint, allowing free setting of the measurement range. On the other hand, because TD-OCT requires mechanical scanning in the depth direction, its measurement speed is low, and measurement repeatability is limited. In this study, a variable optical path length mechanism using a combination of a rotating reflector and a fixed mirror is devised and adopted. Using the rotating mechanism provides reproducibility and stability of measurements. Moreover, since the rotation radius and rotation speed can be freely set in this mechanism, the former determines the measurement range, and the latter fixes the measurement speed, allowing them to be changed freely depending on the target and measurement method. Furthermore, multiple rotating reflectors can be arranged on a disk, increasing the number of measurements per rotation. Table 1 shows the dental OCT applications with their advantages and weaknesses. Except for our prototype, the others are SD-OCT or FD-OCT. They are high-speed, 20k–100k Hz, while the system specifications are fixed with the light source. They are designed for chairside dentistry because of their device size/weight. The portable OCT scanner is designed for home medical care because of its compactness and battery-driven nature.

**Figure 1:**
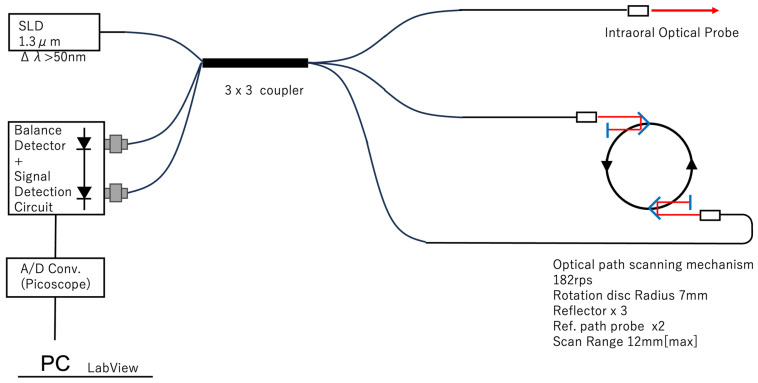
Dental OCT scanner optics. The red lines are light pathes.

**Figure 2:**
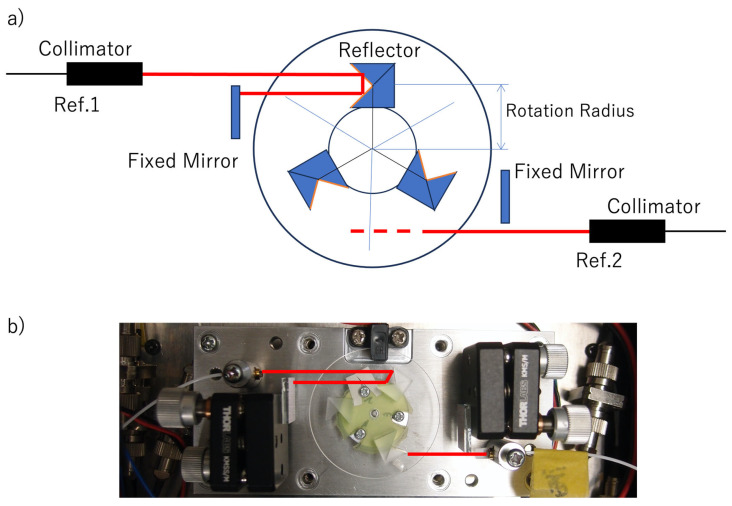
Reference path arrangement on the optical path scanning mechanism. (**a**) Optics arrangement; (**b**) Experimental setup. The red lines are light pathes.

The configuration of the dental TD-OCT scanner is shown in Figure 1. SLD, the OCT light source used in this study, is specially developed to suit this method. Conventional SLDs include a Peltier element for temperature control. This SLD does not have a Peltier element. This consideration is due to the usage of this OCT scanner only for single-use during dental measurement. The light from the SLD source is input into a 3 × 3 fiber coupler. On the input side, one fiber is for input, and two fibers are for detection. On the output side, one fiber is the measurement probe, and the other two fibers are reference fibers. The two reference fibers improve the number of scans per rotation by shifting the timing of light incidence on the reflector. Figure 2 shows an example of this configuration. Figure 2a is the optical arrangement, and Figure 2b is the experimental setup. The reflector’s reference light incidence range is from ±20 to 30 degrees, and multiple reflectors are arranged on a rotating disk. The two detection fibers lead the interference light to a balanced detector. In the case of Figure 2, with three rotating reflectors and two reference fibers, six depth scans (A-scans) are performed in one disk rotation. In this study, the maximum disk rotation speed is 183 rps, resulting in an OCT A-scan measurement speed of 1098 rps. In the early stages of the research, the mechanism of optical path length variation was investigated. It is summarized in Table 2. Interferometric optical ranging enables high-speed scanning with RSOD (Rapid-Scanning Optical Delay) [34]. On the other hand, the Optical interferometry method has a simple configuration with a rotating cube, but scanning is slow [35]. With either method, it is difficult to extend their measurement range to more than several millimeters. In comparison, the method proposed here offers high scalability in both measurement speed and range.

Interference between the probe light and reference light by the 3 × 3 optical fiber coupler alternately occurs between the probe light and Reference 1 or 2 according to the rotation of the reflector. At that time, the interference light causes a phase difference of 4π/3 in the differential detection PDs, respectively, as shown in Equations (1)–(4). Compared to a Mach–Zehnder interferometer using a 2 × 2 fiber coupler, the utilization efficiency of the incident light in the three-branch is 4/9, and the efficiency of the differential due to the phase difference decreases to $\sqrt{3}/2\;=0.866$. In other words, compared to a Michelson interferometer using a 2 × 2 fiber coupler (utilization efficiency of incident light in two branches is 1/2, efficiency of differential due to phase difference is 1), it can be judged that this does not result in a significant loss.

Interference between 1.probe and 2.ref1

(1) $$PD1\to 1.probe+2.ref1={A_{1}}^{2}+{A_{2}}^{2}+2A_{1}A_{2}\cos\left(\phi +\frac{2}{3}\pi \right)$$

(2) $$PD2\to 1.probe+2.ref1={A_{1}}^{2}+{A_{2}}^{2}{+2A}_{1}A_{2}\cos\left(\phi -\frac{2}{3}\pi \right)$$

Interference between 1.probe and 3.ref2

(3) $$PD1\to 1.probe+3.ref2={A_{1}}^{2}+{A_{3}}^{2}+2A_{1}A_{3}\cos\left(\phi +\frac{4}{3}\pi \right)$$

(4) $$PD2\to 1.probe+3.ref2={A_{1}}^{2}+{A_{3}}^{2}+2A_{1}A_{3}\cos\left(\phi +\frac{2}{3}\pi \right)$$

The waveform shaping circuit is developed with the purpose of reducing the load of data sampling by the A/D converter through hardware-based signal processing, and also cutting the computational load of waveform processing after data acquisition. In this waveform shaping circuit, after the I-V conversion circuit detects operation by the PD, the signal passes through the Pre-Amp., BPF, and Main Amp., and is then converted into an envelope of the interferogram by an absolute value circuit to obtain the output waveform. The bandwidth of the BPF is set according to the measurement speed. The waveform measured by the PC after passing through the A/D converter can directly obtain signals of measurement depth and interference light intensity. Since the waveform processing is performed on hardware, the waveform signals can be processed by personal PCs, microcontrollers, etc. Although the dynamic range of this interference light waveform detected by this circuit board is about 45 dB, the sensitivity achieves results equivalent to medical OCT. To improve the dynamic range, it is also possible to secure a dynamic range of up to 100 dB by preparing channels with different amplification factors for the Pre-/Main-Amp and combining the respective amplified signal waveforms [36].

**Figure 3:**
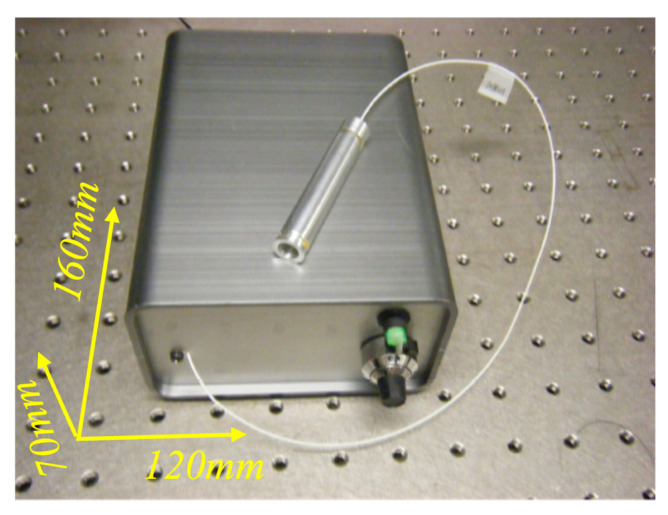
Portable OCT scanner for dental use (minimum size). It is the prototype producted by Phenix-Dent, Co., Ltd., Sano-shi, Japan.

**Figure 4:**
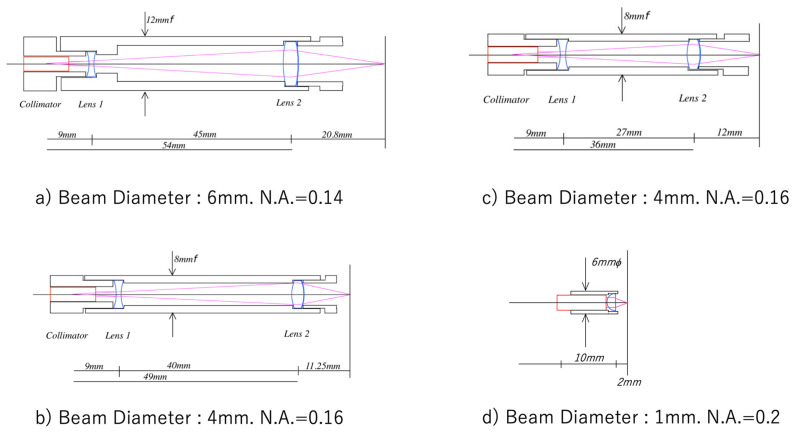
Probe designs for a dental OCT scanner.

Table 3 summarizes the specifications of the dental OCT scanner. A wavelength of 1.3 μm is used, achieving a resolution of 15 μm. The number of measurements realized is 25–1000 scans/s. The measurement range on the OCT is obtained as 12 mm within a rotation radius of 10 mm of the reflector, and a range of ±20°. In practice, the measurement range is narrowed as shown below, depending on the purpose and configuration of the dental probe. On the other hand, the long scanning range is advantageous because it facilitates access to the sample and positioning at the desired measurement location. The external appearance of the portable dental OCT scanner is shown in Figure 3. There are several variations in device development, and this one is configured with the minimum size (the size is slightly different from the specifications in Table 3). On the front of the casing, there is an operation button switch, and measurement starts with a single push. The dial below the switch is for sensitivity adjustment. A stick-shaped optical probe (Figure 4a) extends out.

### 2.2. Probe

**Figure 5:**
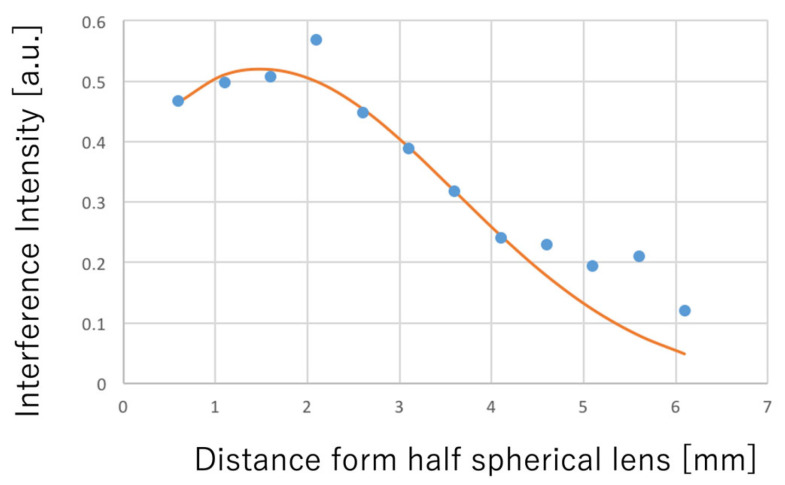
Depth of field of optical probe, Figure 4d for dental OCT scanner. The red curve is the approximated curve calculated by Equation (5).

The dental probe was developed for use inside the oral cavity. By reducing the diameter of the body and focusing light at a close distance, a large NA is achieved. Initially, a mirror was attached to bend the measurement direction by 90 degrees. As measurements progressed, there was an increasing demand to reduce the size of the probe itself, leading to a configuration where a hemispherical lens was attached to the collimator (Figure 4). In reality, the entire probe is as small as 6 mm in diameter and 10 mm in length, allowing it to take any posture freely inside the oral cavity. At this time, the NA became 0.2 at a full-size hemispherical lens, while the incident light from the single-mode fiber is small enough, and the NA is restricted to 0.11–0.14 empirically. The depth of field can be calculated by the following formula.

(5) $$PCF\left(z\right)=\omega_{0}/\left[1+{\left(\frac{z-z_{cf}}{z_{R}}\right)}^{2}\right]$$

where $\omega_{0}$ is the beam waist, z is the depth range, while $z_{cf}$ and $z_{R}$ are the depth location of the focal plane and the Rayleigh length, respectively. The results of the actual measurement of the depth of field are shown in Figure 5. This result is adjusted to match the theoretical values from the previous formula.

## 3. OCT Measurement of Teeth and Gum

**Figure 6:**
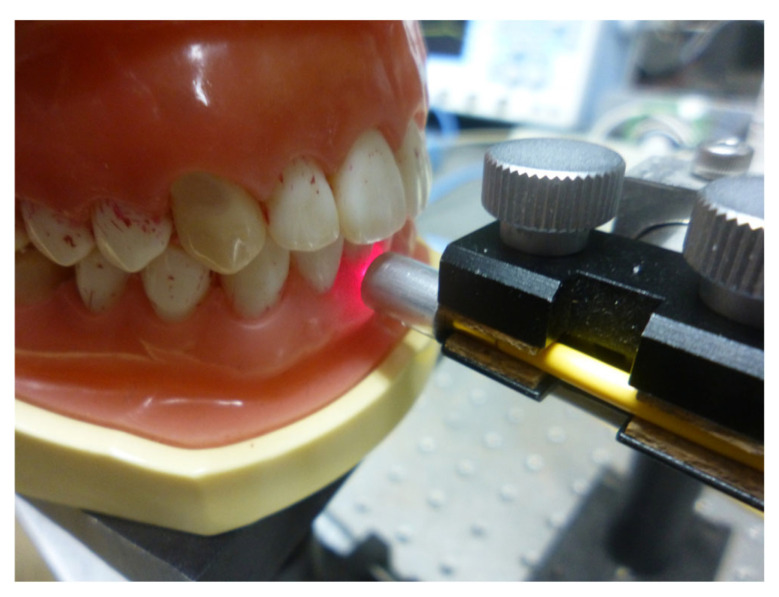
OCT measurement for dental use. The OCT probe brings the target, such as teeth or gum, into the oral cavity.

OCT measurements are performed on dentures, extracted teeth, and natural teeth inside the oral cavity. Figure 6 shows the state of measurement using the developed OCT probe. The probe is brought close to the teeth or gum for measurement. During measurement, the probe does not contact the teeth or the gum itself, and the lens directs the transmitted light through an air layer.

**Figure 7:**
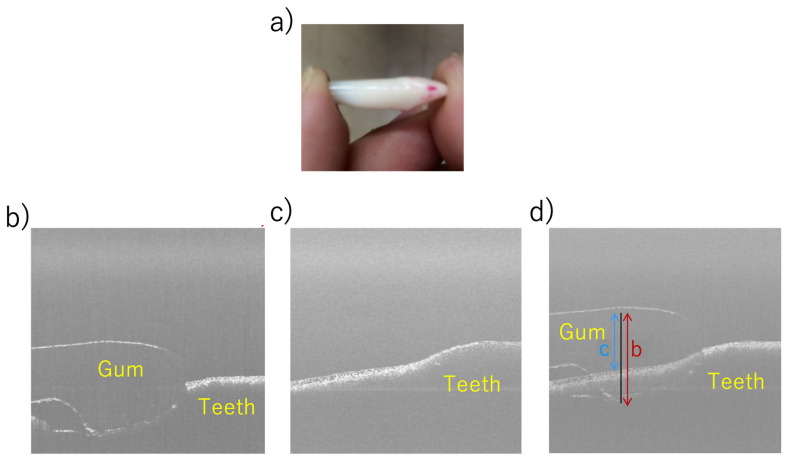
Teeth and gum OCT measurements. The shape of the teeth is largely bent by the gum reflective index. (**a**) Denture; (**b**) Gum and teeth OCT image; (**c**) Teeth OCT image; (**d**) Superimposition of images (**b**,**c**).

Teeth and gums each have their own refractive indices. Therefore, while the surface shape is measured accurately, the interior is distorted by the optical path length (physical distance × refractive index). Figure 7 illustrates this phenomenon. When only the teeth are measured, it can be seen that the tooth surface changes continuously and smoothly (Figure 7c). On the other hand, when the gums cover the teeth, the tooth surface appears distorted due to the optical path length of the gum (Figure 7b). From the superimposition of Figure 7b,c (Figure 7d), the amount of distortion can be understood, and the refractive index of the gums is found to be 1.464. Moreover, this applies when the refractive index of the gingiva is uniform; actual gingiva and teeth vary depending on the location and the degree of damage, and this is reflected in the OCT measurement images.

**Figure 8:**
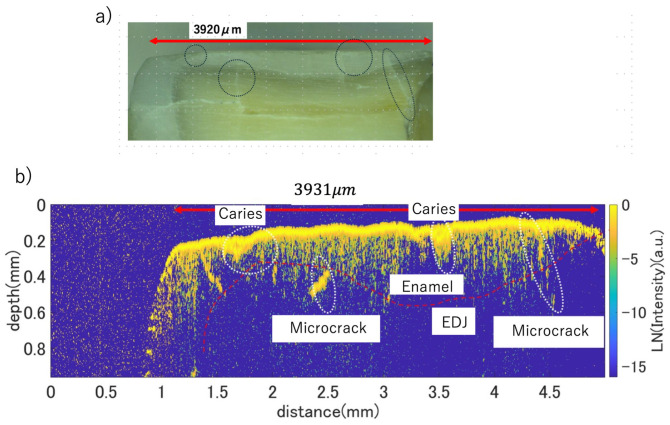
OCT image of front teeth. (**a**) Photograph; (**b**) OCT image. The dashed curve indicates the enamel-dentin Junction.

Figure 8 is an example of measuring caries and microcracks in the enamel layer of an extracted tooth (front teeth). From the surface, since it is also dried, it can be seen as faintly translucent through microscopic observation from the surface. From the OCT image, the extent (width) and depth of the caries can be known, and furthermore, by deriving the attenuation coefficient within the enamel, quantitative evaluation of the caries can be performed. Measurement of the EDJ is also conducted. Compared to the microscopic image cross-section, it is understood that they have the same shape, but the boundary changes due to caries and microcracks. Furthermore, the shape appears distorted due to the refractive index of the enamel, as well as the surface shape and its thickness.

**Figure 9:**
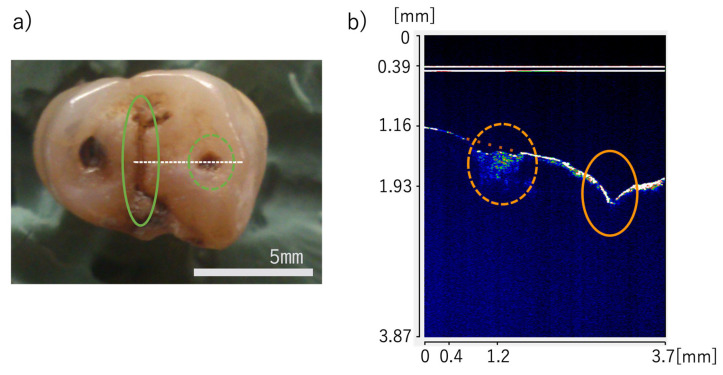
Caries OCT image of molar teeth with caries. (**a**) Molar teeth with caries; (**b**) OCT image. Dashed and solid circles indicate the caries and normal areas.

Figure 9 is another example of caries measurement of a molar tooth. Extracted teeth are easier to diagnose for caries when dry, but it is difficult to identify when wet in the oral cavity. Additionally, the observation posture also causes variability in dentists’ caries diagnosis. In the photo, it was thought that the hole to the right of the central division had a greater degree of caries, but there were no traces of caries in the right hole; rather, the left division area appears to require more serious measures. It can be seen that the carious area returns more scattering from the inside. This is because caries is caused by demineralization, resulting in many small holes that cause increased scattering inside the caries [15]. The depth was about 0.6 mm, and the extent was 0.7 mm.

**Figure 10:**
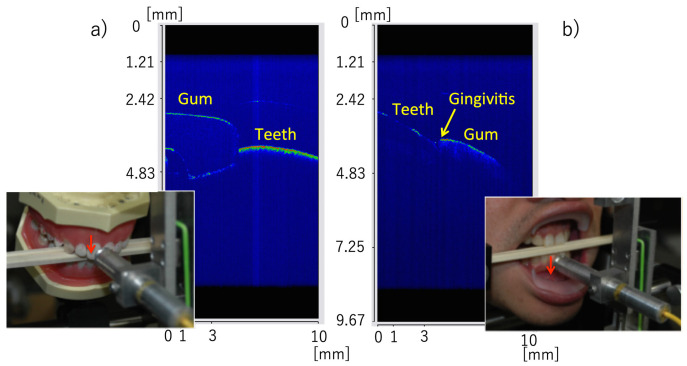
Teeth and gum OCT measurements in the oral cavity. (**a**) Artificial teeth and gum; (**b**) Natural teeth and gum. The red arrows indicate the direction of B-scans.

Figure 10 is an example of measuring inside the oral cavity. The face is fixed, and a comparison with dentures is being made. The gums are healthy, and there is little gap (periodontal pocket) between the teeth and gums. It can be seen that measurements inside the actual oral cavity are being performed equivalently to those with dentures.

## 4. Usage Suggestions

### 4.1. Speed Enhancement

The optical path length variable mechanism in this study can increase the number of rotating reflectors from one to seven. The smaller the rotation radius, the more tolerant it is to the installation angle accuracy of the reflectors. However, the maximum number of rotations per revolution decreases for the same size. Increasing the rotation speed V also raises the beat frequency f, as shown in Equation (6).

(6) f=2V/λ

where λ is the wavelength of SLD. Additionally, since it is a rotational motion, the beat frequency varies within the measurement range. At a beat frequency of 5 MHz, a ±20-degree rotation angle results in about a 1 MHz change. The waveform shaping circuit secures that bandwidth with a BPF (active filter). The filter’s Q value is about 2. When the rotation radius R = 10 mm, the measurement range is 12 mm within a ±20-degree rotation angle range. With a reflector’s rotation radius r = 7 mm, the same measurement range is obtained at ±30 degrees, but the deviation from linear scanning becomes larger. The former has about 1–2%, and the latter about 2–5%. Correction is possible at the reflector position angle, and especially for industrial applications, precision is required for positioning and thickness measurements. In this study, a rotation disk with the reflector’s rotation radius r = 60 mm secures a measurement range of 100 mm and achieves positioning accuracy of 1 μm through correction. The maximum number of measurements achieved is 1000 scans/s with two reference fibers, three rotating reflectors, and a rotation speed of 183 rps. In our experience, the maximum number of reflectors is five.

### 4.2. Expandability

**Figure 11:**
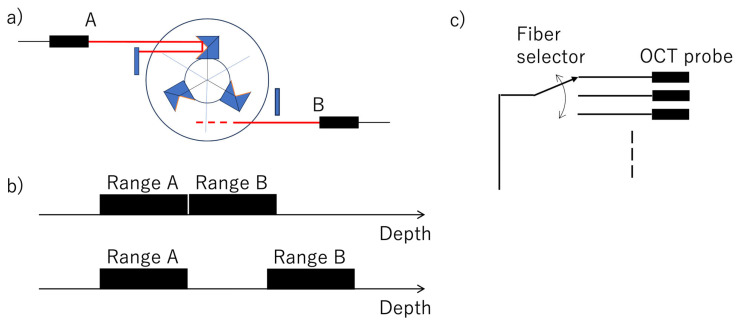
Fiber assembly arrangements. (**a**) Plural ref. fibers and reflectors; (**b**) Range arrangements for continuous range and separate range; (**c**) Probe channel selector.

Expanding the measurement range is also possible by arranging the reference fiber in the optical path length variable mechanism. An example of the arrangement is shown in Figure 11a. The optical path lengths A and B are varied. Changes in optical path length can be made by either the length of the reference fiber or the arrangement of the collimator, allowing two measurement ranges to be arranged continuously or intermittently. As shown in Figure 11b, this can be arranged either continuously (A + B) or intermittently (A and B). Additionally, the probe can be made multi-channel, switching measurements at multiple points with a fiber channel selector. Figure 11c shows this situation, where all probes have the same focal length and are bundled and arranged in a line for multi-point measurement, or probes are placed at multiple desired measurement points with different focal lengths for each. In this case, the optical path length of the reference probe to the rotating reflector is matched accordingly. Switching probes can be performed not only with a channel selector, but also with a 1 × 3 coupler if the light source has sufficient optical power. This is because interference occurs only with the optical path length matched to the reflector. In this study, measurement is realized by switching up to eight channels with a channel selector. Table 4 summarizes examples of specifications for the realized optical path length variable mechanism due to the specified targets (dental, skin, plant measurements, long-range purpose, and high speeds).

## 5. Conclusions

This study reports the development of a portable OCT scanner intended for chairside use. The device size fits within the footprint of a B5 sheet of paper and weighs about 1 kg. Additionally, the intraoral probe measures approximately 6 mm in diameter by 10 mm in length, allowing measurements of teeth and gum within the oral cavity in various postures. Being a Time-Domain type, the measurement speed is slower compared to SD-OCT and SS-OCT, but it achieves a maximum speed of 1000 scans per second, which is sufficient for dental applications. It can also be battery-powered, making it portable enough for use in home visits and other fieldwork. Currently, measurement cases mainly focus on extracted teeth and dentures. A key feature of this TD-OCT scanner is the ability to freely combine measurement range, speed, and light source selection (wavelength, optical output, coherence length, etc.). This allows for high flexibility in probe design. Although its dynamic range is lower than that of medical OCT devices, its sensitivity is nearly equivalent to that of the commercialized medical OCT, and this dental OCT scanner’s specifications have also been applied to measurements of skin and plants. Its multifunctionality offers advantages for industrial needs, enabling measurements tailored to various sample types. The extensibility of TD-OCT lies in its high degree of design flexibility. In particular, with the rotation mechanism of the reflector we proposed, measurement speed and range can be freely adjustable. Furthermore, since the optical probe can be designed to be extremely small, it can be arranged side by side and switched with a selector, allowing for configurations that match the shape of the oral cavity, teeth, and gums. For example, it is conceivable to incorporate the optical probe into a mouthpiece for use.

For dental applications going forward, measurement methods, evaluation techniques, and OCT image processing tailored to specific measurement targets will be further explored.

## Figures and Tables

**Table 1:** Dental OCT applications.

	Portable OCT Scanner	Hand-Held Intraoral OCT	Octina	OCTiX
Product	Phenix Dent (Sano, Japan) Prototype	Medical Laser Center Lübeck (Lübeck, Germany) Prototype	Yoshida Dental MFG (Tokyo, Japan) Commercialized	Huvitz (Anyang-si, Republic of Korea) Commercialized
OCT type	TD-OCT	SD-OCT (Thorlabs GmbH, Bergkirchen, Germany)	SD-OCT	FD-OCT
Device type	Portable	Desktop	Chair Side	Chair Side
Probe type	Fiber type	Gun type	Gun type	Bar type
Advantage	DC-driven Home Care	High speed (20 k–100 kHz)	High speed (50 kHz)	High speed (No info.)
Weakness	Low speed (25–1 kHz)	Hospital Visit	Hospital Visit	Hospital Visit

**Table 2:** Scanning mechanisms of the optical path for TD-OCT.

	Portable OCT Scanner	Interferometric Optical Ranging	Optical Interferometry
Scanning	Rotation of off-axis reflector	Rapid-scanning optical delay (RSOD)	Rotating Cube
speed	25–1000 Hz	1200 Hz	22 Hz (45 ms)
Scanning range	14mm (Variable)	3 mm	1.5 mm

**Table 3:** Specification of portable OCT scanner for dental use.

SLD	Anritsu AS3E113HJ10M (Anritsu, Singapore) $\lambda_{0}=1.3\mu m,\Delta \lambda >50\;nm,3\;mW\;[max]$
Coherent length	15μm
Scan speed	1000 rps [max]
Scanning range	12 mm
Probe size	8 mmφ × 10 mm
Probe depth of field	3 mm
OCT size	200 mm × 150 mm × 100 mm
Weight	1 kg

**Table 4:** Arrangements for rotational TD-OCT scanner.

	Reflector	Reference Fiber	Radius [mm]	Rotation [rps]	Range [mm]	Scan Speed [scan/s]
Dental Skin Plant	1	2	10	25	12	25
Long range	1	1	60	4	>80	4
High speed	3	2	10	125	12	750
High speed	3	2	7	183	>14	>1000

## Data Availability

The data that support the findings of this study are available from the corresponding author upon reasonable request.
